# 4,4′-Bipyridine–3,3′-disulfanediyl­bis­(1*H*-1,2,4-triazole-5-amine) (1/1)

**DOI:** 10.1107/S1600536812042742

**Published:** 2012-10-20

**Authors:** Wei Yang, Qi-Ming Qiu, Qiong-Hua Jin, Cun-Lin Zhang

**Affiliations:** a Department of Chemistry, Capital Normal University, Beijing 100048, People’s Republic of China; b Key Laboratory of Terahertz Optoelectronics, Ministry of Education, Department of Physics, Capital Normal University, Beijing 100048, People’s Republic of China

## Abstract

In the title 1:1 adduct, C_10_H_8_N_2_·C_4_H_6_N_8_S_2_·, the components are connected through N—H⋯N hydrogen bonds, leading to a two-dimensional structure. The C—S—S—C torsion angle is −83.6 (1)°. The dihedral angle between pyridine rings is 1.86 (15)°.

## Related literature

For structures containing 1*H*-1,2,4-triazole-5-amine-3-thiol­ate, see: Aldoshin *et al.* (2003 ▶); Hao *et al.* (2010 ▶); Rakova *et al.* (2003 ▶). For related structures, see: Brito *et al.* (2007 ▶); Deng *et al.* (2005 ▶). 
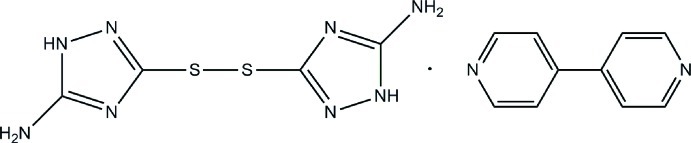


## Experimental

### 

#### Crystal data

C_10_H_8_N_2_·C_4_H_6_N_8_S_2_
*M* *_r_* = 386.47
Triclinic, 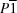

*a* = 9.324 (1) Å
*b* = 9.4540 (11) Å
*c* = 11.3840 (13) Å
α = 109.560 (2)°
β = 104.089 (1)°
γ = 105.627 (1)°
*V* = 846.86 (17) Å^3^
*Z* = 2
Mo *K*α radiation
μ = 0.34 mm^−1^
*T* = 298 K
0.35 × 0.30 × 0.21 mm

#### Data collection

Bruker SMART CCD area-detector diffractometer
Absorption correction: multi-scan (*SADABS*; Bruker, 2007 ▶) *T*_min_ = 0.891, *T*_max_ = 0.933
4439 measured reflections
2952 independent reflections
2121 reflections with *I* > 2σ(*I*)
*R*_int_ = 0.019

#### Refinement

*R*[*F* ^2^ > 2σ(*F* ^2^)] = 0.043
*wR*(*F* ^2^) = 0.106
*S* = 1.04
2952 reflections
235 parameters
H-atom parameters constrained
Δρ_max_ = 0.26 e Å^−3^
Δρ_min_ = −0.26 e Å^−3^

### 

Data collection: *SMART* (Bruker, 2007 ▶); cell refinement: *SAINT-Plus* (Bruker, 2007 ▶); data reduction: *SAINT-Plus*; program(s) used to solve structure: *SHELXS97* (Sheldrick, 2008 ▶); program(s) used to refine structure: *SHELXL97* (Sheldrick, 2008 ▶); molecular graphics: *SHELXTL* (Sheldrick, 2008 ▶); software used to prepare material for publication: *SHELXTL*.

## Supplementary Material

Crystal structure: contains datablock(s) global, I. DOI: 10.1107/S1600536812042742/qm2081sup1.cif

Structure factors: contains datablock(s) I. DOI: 10.1107/S1600536812042742/qm2081Isup2.hkl

Supplementary material file. DOI: 10.1107/S1600536812042742/qm2081Isup3.cml

Additional supplementary materials:  crystallographic information; 3D view; checkCIF report

## Figures and Tables

**Table 1:** Hydrogen-bond geometry (Å, °)

*D*—H⋯*A*	*D*—H	H⋯*A*	*D*⋯*A*	*D*—H⋯*A*
N2—H2⋯N1^i^	0.86	2.22	2.850 (3)	131
N4—H4*A*⋯N5^i^	0.86	2.29	3.058 (3)	149
N4—H4*B*⋯N10^ii^	0.86	2.18	2.977 (3)	154
N6—H6⋯N9^iii^	0.86	2.04	2.867 (3)	162
N8—H8*A*⋯N3^iv^	0.86	2.33	3.137 (3)	156
N8—H8*B*⋯N7^v^	0.86	2.22	3.068 (3)	167

Symmetry codes: (i) 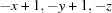; (ii) 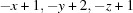; (iii) 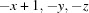; (iv) 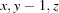; (v) 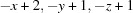.

**Fig. 1.**
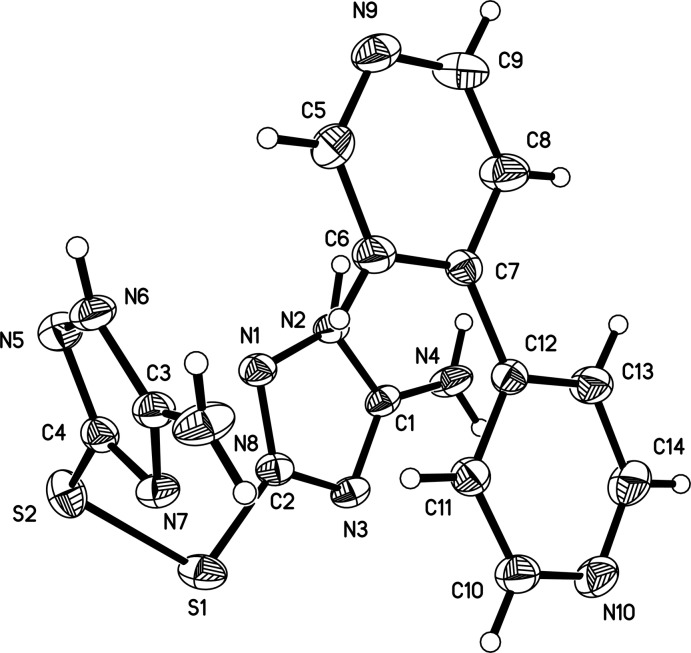
The molecular entities of the title compound, showing the atom-numbering scheme with displacement ellipsoids drawn at the 50% probability level.

**Fig. 2.**
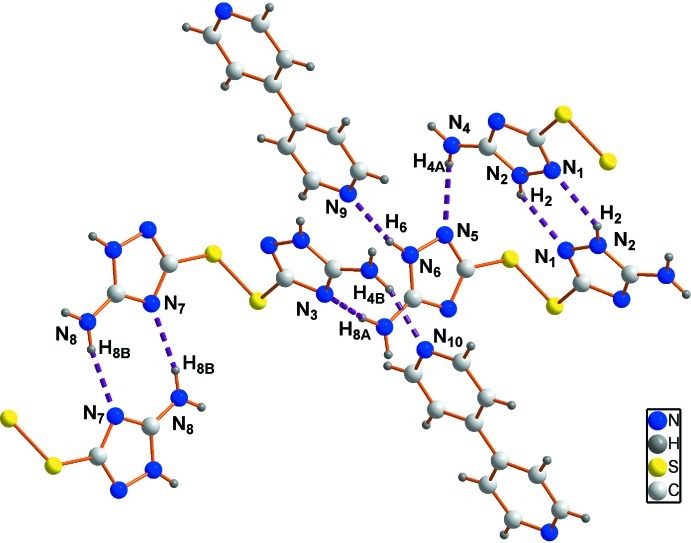
Intermolecular N—H···N hydrogen bonds.

**Fig. 3.**
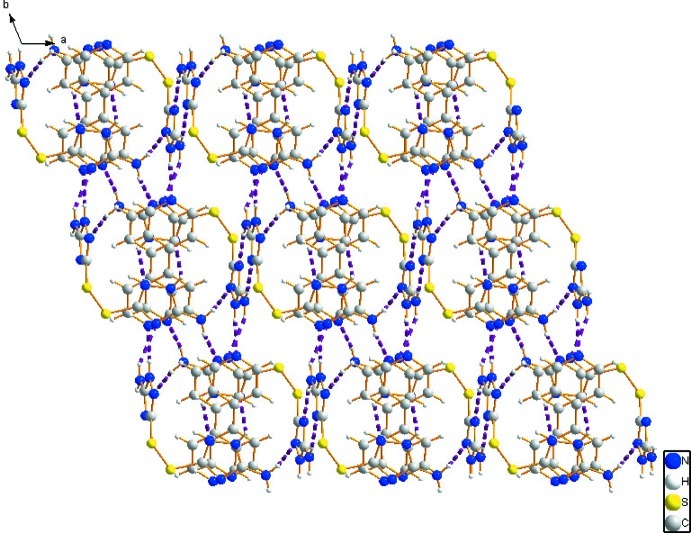
Intermolecular N—H···N hydrogen bonds.

## supplementary crystallographic information

### Comment

The design and synthesis of novel inorganic-organic hybrid coordination complexes have attracted the attention of many chemists in recent years. So far, there are very few literature reports of structures containing 1*H*-1,2,4-triazole-5-amine-3-thiolate (Rakova *et al.*2003; Hao *et al.*, 2010; Aldoshin *et al.*, 2003). We are interested in synthesizing new transition metal complexes containing 5-AMT. The title co-crystal was unexpectedly obtained in the course of synthesizing 5-AMT-Ni(II) complexes.

The molecular structure of the co-crystal is shown in Fig.1. The title compound is triclinic in the P-1 space group. C_4_H_6_N_8_S_2_.C_10_H_8_N_2_ contains two 5-AMT units linked by an S—S disulfide bridge. The C—S—S—C torsion angle is 83.6 (1)°. This value is close to that of 81.9 (1)° determined for 5,5'-Dithiobis(1-phenyl-1*H*-tetrazole) (Brito *et al.*, 2007). The 4,4'-bipyridine molecule is connected to a C_4_H_6_N_8_S_2_ molecule through N—H···N hydrogen bonds, which are similar to those in the co-crystal of C_10_H_8_N_2_.2C_2_H_3_N_3_S_2_ (Deng *et al.*, 2005). Further N—H···N hydrogen bonds between C_4_H_6_N_8_S_2_ molecules leads to a two-dimensional network (Fig.2 and Fig.3). There are face-to-face πi-πi stacking interactions between the 4,4'-bipyridine and triazole rings, the centroid-centroid distance is 3.630 Å.

### Experimental

The title co-crystal has been prepared by adding 5-AMT(1.8 mmol), sodium hydroxide(1.2 mmol) and 4,4'-bipyridine(1.0 mmol) into a stirred mixture of CH_3_OH (7 mL) and H_2_O (5 mL) containing Ni(NO_3_)_2_.6H_2_O (1.0 mmol). The mixture was refluxed for 5 h and then allowed to cool to ambient temperature. The filtrate was evaporated slowly at room temperature for 3 days to yield yellow crystalline products.

### Refinement

Metal atom centers were located from the E-maps and other non-hydrogen atoms were located in successive difference Fourier syntheses. The final refinements were performed by full matrix least-squares methods with anisotropic thermal parameters for non-hydrogen atoms on F^2^.

The final refinements were performed by full martrix least-squares methods with anisotropic thermal parameters for non-hydrogen atoms on F^2^. All hydrogen atoms were located in the calculated sites and included in the final refinement in the riding model approximation with displacement parameters derived from the parent atoms to which they were bonded (U_iso_(H) = 1.2Ueq). C-H hydrogen atoms (aromatic) were included with distance set to 0.93Å and amide N-H hydrogen atoms were included with distance set to 0.86Å.

### Crystal data

**Table:**

C_10_H_8_N_2_·C_4_H_6_N_8_S_2_	*Z* = 2
*M**_r_* = 386.47	*F*(000) = 400
Triclinic, *P*1	*D*_x_ = 1.516 Mg m^−^^3^
*a* = 9.324 (1) Å	Mo *K*α radiation, λ = 0.71073 Å
*b* = 9.4540 (11) Å	Cell parameters from 1617 reflections
*c* = 11.3840 (13) Å	θ = 2.4–26.4°
α = 109.560 (2)°	µ = 0.34 mm^−^^1^
β = 104.089 (1)°	*T* = 298 K
γ = 105.627 (1)°	Block, yellow
*V* = 846.86 (17) Å^3^	0.35 × 0.30 × 0.21 mm

### Data collection

**Table:**

Bruker SMART CCD area-detector diffractometer	2952 independent reflections
Radiation source: fine-focus sealed tube	2121 reflections with *I* > 2σ(*I*)
Graphite monochromator	*R*_int_ = 0.019
phi and ω scans	θ_max_ = 25.0°, θ_min_ = 2.4°
Absorption correction: multi-scan (*SADABS*; Bruker, 2007)	*h* = −10→11
*T*_min_ = 0.891, *T*_max_ = 0.933	*k* = −6→11
4439 measured reflections	*l* = −13→13

### Refinement

**Table:**

Refinement on *F*^2^	Primary atom site location: structure-invariant direct methods
Least-squares matrix: full	Secondary atom site location: difference Fourier map
*R*[*F*^2^ > 2σ(*F*^2^)] = 0.043	Hydrogen site location: inferred from neighbouring sites
*wR*(*F*^2^) = 0.106	H-atom parameters constrained
*S* = 1.04	*w* = 1/[σ^2^(*F*_o_^2^) + (0.0426*P*)^2^ + 0.3426*P*] where *P* = (*F*_o_^2^ + 2*F*_c_^2^)/3
2952 reflections	(Δ/σ)_max_ < 0.001
235 parameters	Δρ_max_ = 0.26 e Å^−^^3^
0 restraints	Δρ_min_ = −0.26 e Å^−^^3^

### Special details

**Table:**

Geometry. All e.s.d.'s (except the e.s.d. in the dihedral angle between two l.s. planes) are estimated using the full covariance matrix. The cell e.s.d.'s are taken into account individually in the estimation of e.s.d.'s in distances, angles and torsion angles; correlations between e.s.d.'s in cell parameters are only used when they are defined by crystal symmetry. An approximate (isotropic) treatment of cell e.s.d.'s is used for estimating e.s.d.'s involving l.s. planes.
Refinement. Refinement of *F*^2^ against ALL reflections. The weighted *R*-factor *wR* and goodness of fit *S* are based on *F*^2^, conventional *R*-factors *R* are based on *F*, with *F* set to zero for negative *F*^2^. The threshold expression of *F*^2^ > σ(*F*^2^) is used only for calculating *R*-factors(gt) *etc*. and is not relevant to the choice of reflections for refinement. *R*-factors based on *F*^2^ are statistically about twice as large as those based on *F*, and *R*- factors based on ALL data will be even larger.

### Fractional atomic coordinates and isotropic or equivalent isotropic displacement parameters (Å^2^)

**Table:**

	*x*	*y*	*z*	*U*_iso_*/*U*_eq_	
N1	0.6415 (3)	0.6418 (2)	0.1281 (2)	0.0365 (5)	
N2	0.4984 (3)	0.6630 (3)	0.1020 (2)	0.0367 (5)	
H2	0.4095	0.5917	0.0394	0.044*	
N3	0.6698 (3)	0.8909 (2)	0.2725 (2)	0.0368 (5)	
N4	0.3976 (3)	0.8634 (3)	0.1817 (2)	0.0499 (7)	
H4A	0.3040	0.8023	0.1210	0.060*	
H4B	0.4143	0.9587	0.2385	0.060*	
N5	0.8542 (3)	0.3413 (3)	0.1018 (2)	0.0411 (6)	
N6	0.8400 (3)	0.2361 (3)	0.1625 (2)	0.0398 (6)	
H6	0.7998	0.1318	0.1206	0.048*	
N7	0.9544 (2)	0.4807 (2)	0.3285 (2)	0.0346 (5)	
N8	0.9016 (3)	0.2543 (3)	0.3827 (2)	0.0505 (7)	
H8A	0.8650	0.1503	0.3539	0.061*	
H8B	0.9406	0.3155	0.4674	0.061*	
N9	0.2801 (3)	0.0985 (3)	0.0248 (2)	0.0488 (6)	
N10	0.5992 (3)	0.8714 (3)	0.5836 (2)	0.0518 (7)	
S1	0.94031 (9)	0.82733 (9)	0.31441 (8)	0.0479 (2)	
S2	0.98658 (9)	0.65858 (9)	0.17888 (8)	0.0474 (2)	
C1	0.5172 (3)	0.8101 (3)	0.1876 (3)	0.0342 (6)	
C2	0.7366 (3)	0.7806 (3)	0.2306 (3)	0.0345 (6)	
C3	0.8980 (3)	0.3206 (3)	0.2961 (3)	0.0349 (6)	
C4	0.9235 (3)	0.4833 (3)	0.2058 (3)	0.0338 (6)	
C5	0.4333 (4)	0.1698 (4)	0.1025 (3)	0.0540 (8)	
H5	0.4996	0.1149	0.0828	0.065*	
C6	0.5013 (3)	0.3195 (3)	0.2107 (3)	0.0478 (8)	
H6A	0.6104	0.3626	0.2611	0.057*	
C7	0.4085 (3)	0.4057 (3)	0.2443 (3)	0.0328 (6)	
C8	0.2490 (4)	0.3329 (3)	0.1624 (3)	0.0526 (8)	
H8	0.1800	0.3855	0.1789	0.063*	
C9	0.1915 (4)	0.1821 (4)	0.0559 (3)	0.0565 (9)	
H9	0.0832	0.1364	0.0027	0.068*	
C10	0.6900 (4)	0.7885 (3)	0.5540 (3)	0.0473 (7)	
H10	0.7973	0.8336	0.6097	0.057*	
C11	0.6350 (3)	0.6395 (3)	0.4456 (3)	0.0391 (7)	
H11	0.7049	0.5881	0.4293	0.047*	
C12	0.4754 (3)	0.5667 (3)	0.3612 (3)	0.0326 (6)	
C13	0.3814 (4)	0.6530 (3)	0.3920 (3)	0.0490 (8)	
H13	0.2735	0.6108	0.3388	0.059*	
C14	0.4475 (4)	0.8020 (4)	0.5018 (3)	0.0565 (9)	
H14	0.3808	0.8573	0.5195	0.068*	

### Atomic displacement parameters (Å^2^)

**Table:**

	*U*^11^	*U*^22^	*U*^33^	*U*^12^	*U*^13^	*U*^23^
N1	0.0370 (13)	0.0262 (12)	0.0393 (13)	0.0107 (10)	0.0112 (11)	0.0092 (10)
N2	0.0333 (13)	0.0298 (12)	0.0337 (12)	0.0087 (10)	0.0081 (10)	0.0041 (10)
N3	0.0449 (14)	0.0256 (11)	0.0312 (12)	0.0104 (10)	0.0089 (11)	0.0083 (10)
N4	0.0478 (15)	0.0401 (14)	0.0402 (14)	0.0223 (12)	0.0023 (12)	−0.0016 (11)
N5	0.0422 (14)	0.0398 (14)	0.0339 (13)	0.0141 (11)	0.0088 (11)	0.0123 (11)
N6	0.0417 (14)	0.0273 (12)	0.0344 (13)	0.0096 (10)	0.0073 (11)	0.0026 (10)
N7	0.0365 (13)	0.0254 (11)	0.0327 (12)	0.0096 (10)	0.0070 (10)	0.0078 (10)
N8	0.0713 (18)	0.0257 (12)	0.0387 (14)	0.0086 (12)	0.0105 (13)	0.0103 (11)
N9	0.0640 (18)	0.0304 (13)	0.0403 (14)	0.0096 (13)	0.0162 (13)	0.0104 (11)
N10	0.0624 (18)	0.0364 (14)	0.0462 (15)	0.0167 (13)	0.0216 (14)	0.0067 (12)
S1	0.0404 (4)	0.0315 (4)	0.0540 (5)	0.0084 (3)	0.0038 (4)	0.0120 (3)
S2	0.0447 (5)	0.0507 (5)	0.0620 (5)	0.0224 (4)	0.0264 (4)	0.0334 (4)
C1	0.0440 (17)	0.0257 (14)	0.0290 (14)	0.0112 (12)	0.0123 (13)	0.0099 (12)
C2	0.0381 (16)	0.0252 (14)	0.0362 (15)	0.0086 (12)	0.0109 (13)	0.0133 (12)
C3	0.0321 (15)	0.0282 (14)	0.0351 (15)	0.0093 (12)	0.0081 (12)	0.0078 (12)
C4	0.0274 (14)	0.0332 (15)	0.0368 (15)	0.0130 (12)	0.0082 (12)	0.0121 (13)
C5	0.056 (2)	0.0391 (17)	0.060 (2)	0.0200 (16)	0.0279 (18)	0.0077 (15)
C6	0.0368 (17)	0.0380 (16)	0.0511 (18)	0.0121 (13)	0.0125 (14)	0.0036 (14)
C7	0.0347 (15)	0.0286 (14)	0.0354 (15)	0.0093 (12)	0.0134 (12)	0.0158 (12)
C8	0.0428 (18)	0.0387 (17)	0.057 (2)	0.0166 (14)	0.0068 (15)	0.0065 (15)
C9	0.0483 (19)	0.0402 (18)	0.055 (2)	0.0074 (15)	0.0001 (16)	0.0121 (16)
C10	0.0466 (18)	0.0402 (17)	0.0425 (17)	0.0099 (14)	0.0116 (15)	0.0120 (14)
C11	0.0386 (16)	0.0334 (15)	0.0423 (16)	0.0137 (13)	0.0150 (13)	0.0129 (13)
C12	0.0360 (15)	0.0265 (13)	0.0347 (14)	0.0105 (12)	0.0133 (12)	0.0135 (12)
C13	0.0392 (17)	0.0426 (17)	0.0503 (18)	0.0173 (14)	0.0116 (14)	0.0052 (14)
C14	0.062 (2)	0.0481 (19)	0.057 (2)	0.0318 (17)	0.0263 (18)	0.0082 (16)

### Geometric parameters (Å, º)

**Table:**

N1—C2	1.309 (3)	N10—C10	1.328 (4)
N1—N2	1.378 (3)	S1—C2	1.760 (3)
N2—C1	1.339 (3)	S1—S2	2.0392 (11)
N2—H2	0.8600	S2—C4	1.757 (3)
N3—C1	1.339 (3)	C5—C6	1.375 (4)
N3—C2	1.366 (3)	C5—H5	0.9300
N4—C1	1.338 (3)	C6—C7	1.376 (4)
N4—H4A	0.8600	C6—H6A	0.9300
N4—H4B	0.8600	C7—C8	1.378 (4)
N5—C4	1.310 (3)	C7—C12	1.485 (3)
N5—N6	1.385 (3)	C8—C9	1.380 (4)
N6—C3	1.341 (3)	C8—H8	0.9300
N6—H6	0.8600	C9—H9	0.9300
N7—C3	1.343 (3)	C10—C11	1.382 (4)
N7—C4	1.366 (3)	C10—H10	0.9300
N8—C3	1.333 (3)	C11—C12	1.386 (4)
N8—H8A	0.8600	C11—H11	0.9300
N8—H8B	0.8600	C12—C13	1.378 (4)
N9—C9	1.319 (4)	C13—C14	1.380 (4)
N9—C5	1.321 (4)	C13—H13	0.9300
N10—C14	1.322 (4)	C14—H14	0.9300
			
C2—N1—N2	101.2 (2)	N7—C4—S2	125.08 (19)
C1—N2—N1	110.6 (2)	N9—C5—C6	124.5 (3)
C1—N2—H2	124.7	N9—C5—H5	117.8
N1—N2—H2	124.7	C6—C5—H5	117.8
C1—N3—C2	101.9 (2)	C5—C6—C7	120.1 (3)
C1—N4—H4A	120.0	C5—C6—H6A	119.9
C1—N4—H4B	120.0	C7—C6—H6A	119.9
H4A—N4—H4B	120.0	C6—C7—C8	115.7 (2)
C4—N5—N6	101.7 (2)	C6—C7—C12	122.3 (2)
C3—N6—N5	110.1 (2)	C8—C7—C12	122.0 (2)
C3—N6—H6	125.0	C7—C8—C9	120.1 (3)
N5—N6—H6	125.0	C7—C8—H8	120.0
C3—N7—C4	102.4 (2)	C9—C8—H8	120.0
C3—N8—H8A	120.0	N9—C9—C8	124.3 (3)
C3—N8—H8B	120.0	N9—C9—H9	117.9
H8A—N8—H8B	120.0	C8—C9—H9	117.9
C9—N9—C5	115.4 (2)	N10—C10—C11	124.1 (3)
C14—N10—C10	115.7 (2)	N10—C10—H10	118.0
C2—S1—S2	102.20 (9)	C11—C10—H10	118.0
C4—S2—S1	104.74 (9)	C10—C11—C12	119.9 (2)
N4—C1—N2	122.7 (2)	C10—C11—H11	120.1
N4—C1—N3	127.5 (2)	C12—C11—H11	120.1
N2—C1—N3	109.8 (2)	C13—C12—C11	115.9 (2)
N1—C2—N3	116.6 (2)	C13—C12—C7	121.6 (2)
N1—C2—S1	123.3 (2)	C11—C12—C7	122.4 (2)
N3—C2—S1	120.13 (19)	C12—C13—C14	120.1 (3)
N8—C3—N6	124.6 (2)	C12—C13—H13	120.0
N8—C3—N7	125.6 (2)	C14—C13—H13	120.0
N6—C3—N7	109.8 (2)	N10—C14—C13	124.3 (3)
N5—C4—N7	116.1 (2)	N10—C14—H14	117.8
N5—C4—S2	118.5 (2)	C13—C14—H14	117.8
			
C2—N1—N2—C1	0.6 (3)	S1—S2—C4—N7	−44.3 (2)
C4—N5—N6—C3	1.1 (3)	C9—N9—C5—C6	0.8 (5)
C2—S1—S2—C4	−83.57 (13)	N9—C5—C6—C7	0.1 (5)
N1—N2—C1—N4	178.1 (2)	C5—C6—C7—C8	−1.0 (4)
N1—N2—C1—N3	−0.1 (3)	C5—C6—C7—C12	179.1 (3)
C2—N3—C1—N4	−178.5 (3)	C6—C7—C8—C9	1.0 (4)
C2—N3—C1—N2	−0.4 (3)	C12—C7—C8—C9	−179.1 (3)
N2—N1—C2—N3	−0.9 (3)	C5—N9—C9—C8	−0.8 (5)
N2—N1—C2—S1	178.47 (19)	C7—C8—C9—N9	0.0 (5)
C1—N3—C2—N1	0.8 (3)	C14—N10—C10—C11	0.3 (5)
C1—N3—C2—S1	−178.55 (19)	N10—C10—C11—C12	−1.0 (5)
S2—S1—C2—N1	17.6 (2)	C10—C11—C12—C13	1.0 (4)
S2—S1—C2—N3	−163.10 (19)	C10—C11—C12—C7	−179.1 (3)
N5—N6—C3—N8	−179.9 (3)	C6—C7—C12—C13	178.7 (3)
N5—N6—C3—N7	−1.2 (3)	C8—C7—C12—C13	−1.3 (4)
C4—N7—C3—N8	179.5 (3)	C6—C7—C12—C11	−1.3 (4)
C4—N7—C3—N6	0.8 (3)	C8—C7—C12—C11	178.8 (3)
N6—N5—C4—N7	−0.6 (3)	C11—C12—C13—C14	−0.4 (4)
N6—N5—C4—S2	173.25 (17)	C7—C12—C13—C14	179.7 (3)
C3—N7—C4—N5	−0.1 (3)	C10—N10—C14—C13	0.4 (5)
C3—N7—C4—S2	−173.50 (19)	C12—C13—C14—N10	−0.3 (5)
S1—S2—C4—N5	142.40 (19)		

### Hydrogen-bond geometry (Å, º)

**Table:**

*D*—H···*A*	*D*—H	H···*A*	*D*···*A*	*D*—H···*A*
N2—H2···N1^i^	0.86	2.22	2.850 (3)	131
N4—H4*A*···N5^i^	0.86	2.29	3.058 (3)	149
N4—H4*B*···N10^ii^	0.86	2.18	2.977 (3)	154
N6—H6···N9^iii^	0.86	2.04	2.867 (3)	162
N8—H8*A*···N3^iv^	0.86	2.33	3.137 (3)	156
N8—H8*B*···N7^v^	0.86	2.22	3.068 (3)	167

Symmetry codes: (i) −*x*+1, −*y*+1, −*z*; (ii) −*x*+1, −*y*+2, −*z*+1; (iii) −*x*+1, −*y*, −*z*; (iv) *x*, *y*−1, *z*; (v) −*x*+2, −*y*+1, −*z*+1.
